# Intersectoral cooperation between university hospitals and physicians in private practice in Germany– where the potential for optimization lies

**DOI:** 10.1186/s12913-024-10963-8

**Published:** 2024-04-22

**Authors:** R. M. Waeschle, T. Epperlein, I. Demmer, E. Hummers, Q. Quintel

**Affiliations:** 1 Department of Anaesthesiology, University Medical Centre Göttingen, Robert-Koch-Str. 40, 37099 Göttingen, Germany; 2 Department of General Practice, University Medical Centre Göttingen, Göttingen, Germany

**Keywords:** Collaboration, Intersectoral, Optimization potential, University hospitals, Private practices

## Abstract

**Background:**

Intersectoral cooperation between physicians in private practice and hospitals is highly relevant for ensuring the quality of medical care. However, the experiences and potential for optimization at this interface from the perspective of physicians in private practice have not yet been systematically investigated. The aim of this questionnaire survey was to record participants’ experiences with regard to cooperation with university hospitals and to identify the potential for optimizing intersectoral cooperation.

**Methods:**

We performed a prospective cross-sectional study using an online survey among practising physicians of all disciplines offering ambulatory care in Germany. The link to a 41-item questionnaire was sent via mail using a commercial mail distributor in which 1095 practising physicians participated. Baseline statistics were performed with SurveyMonkey and Excel.

**Results:**

A total of 70.6%/722 of the responding physicians in private practice rated cooperation with university hospitals as satisfactory. Satisfaction with the quality of treatment was confirmed by 87.2%/956 of the physicians. The subjectively perceived complication rate in patient care was assessed as rare (80.9%/886). However, the median waiting time for patients in the inpatient discharge letter was 4 weeks. The accessibility of medical contact persons was rated as rather difficult by 52.6%/577 of the physicians. A total of 48.6%/629 of the participants considered better communication as an equal partner to be an important potential for optimization. Likewise, 65.2%/714 participants wished for closer cooperation in pre- and/or post inpatient care.

**Conclusion:**

The following optimization potentials were identified: timely discharge letters, clear online presentations of clinical contacts, improved accessibility by telephone, introduction or further development of a referral portal, regular intersectoral training and/or “get-togethers”, regular surveys of general practitioners and implementation of resulting measures, further development of cross-sectoral communication channels and strengthening of hospital IT.

**Supplementary Information:**

The online version contains supplementary material available at 10.1186/s12913-024-10963-8.

## Introduction

Intersectoral cooperation between physicians in private practice and hospitals is highly relevant for ensuring the quality of medical care. Inadequate communication during discharge increases the risk of deficits in care [1], such as incorrect medication adjustments in further outpatient care [2, 3] and rehospitalization within a few weeks [4–6]. This is particularly important because, in Germany, in contrast to that in other countries, the division of the health care system into outpatient and inpatient sectors is very inflexible.

Moreover, in 30%-80% of admissions, the referring physician decides which hospital the patient will be admitted to [7–9]. Thus, this interface is also an important economic factor for hospitals. Accordingly, referral management and the improvement of this collaboration are repeatedly discussed in connection with revenue optimization and market development in hospitals [10].

In this context, surveys of hospitals regarding referral management and referral relationship management have been conducted [10, 11]. In addition, studies have asked patients about their experiences at the interfaces between the sectors [8, 12–14]. However, local surveys of collaboration between office-based physicians and hospitals have been conducted only sporadically—often monocentrically—with a primary focus on discharge letters [15–21]. One study evaluated general practitioner (GP) satisfaction with cooperation with other health care providers in cancer care and evaluated which variables are associated with greater satisfaction [22]. However, a national survey on the opinions of referring physicians of different outpatient specialities regarding cooperation with university hospitals in general has not yet been conducted in Germany.

The aim of this survey of physicians in private practice was therefore to collect semiquantitative data on their experiences regarding collaboration with university hospitals and the possible potential for optimization.

## Materials and methods

### Study design

The survey was designed as a national, cross-sectional survey of physicians in private practice in Germany. Both general practitioners and specialists were included. The survey primarily focused on cooperation with university hospitals. The questions focused on physicians’ perceptions of cooperation, the quality of treatment and the subjective comparison with other hospitals, including reasons for referral or nonreferral to the respective hospitals.

### Questionnaire

A representative of a university medical board, medical staff members of the University Medical Centre Göttingen, a practising internal medicine specialist, and a former member of the Association of Statutory Health Insurance Physicians of Hesse were responsible for the selection of questions. Physicians in private practice and staff members of the Department of General Practice at the University Medical Centre Göttingen piloted and optimized the resulting questionnaire.

The questionnaire included 41 questions on the following aspects:

- The individuals (age, sex, work experience, clinical focus, etc.)
- Current cooperation with the respective university hospital (waiting time for admission, waiting for the discharge letter, accessibility of colleagues, overall satisfaction with the cooperation, information about complications, etc.)
- Subjective perceptions of the treatment quality of the university hospital (satisfaction with treatment results, with information about the clinical stay of the patient, etc.)
- Subjective impressions regarding the development of cooperation and the possible potential for improvement.
- Comparison of subjective perceptions regarding collaboration between the university hospital and the nonuniversity hospital for which referrals are most frequent.

The questions were specifically directed towards cooperation with the respective university hospital to achieve better objectivity of the data evaluation. The collaboration with the department of the university hospital to which the resident physicians referred most frequently was addressed.

There were dichotomous questions, questions with 4-level and 6-level ordinal scales and questions with multiple answers. The time required for completion was approximately 15 min. The complete questionnaire is available in the supplemental material.

### Survey and email distribution list

All 17 Associations of Statutory Health Insurance Physicians (KVs) at the state level were informed about this survey in advance and sent the questionnaire for information. Seven KVs agreed to inform their members about the survey and to ask for their participation. Ten KVs agreed to the survey but decided not to inform their members.

The anonymous survey started using the online platform SurveyMonkey (Momentive Europe UC, Ireland) on February 14, 2019. An email containing a link to complete a single online questionnaire was sent. The email distribution list, commercially acquired from the company “Adress Publisher”, included 103,836 email addresses, of which 60,150 email addresses remained for practising human physicians throughout Germany after thorough quality control. Quality control included the search for invalid e-mail addresses by filtering email addresses, including the terms a.o. “physio”, “ergo”, “san”, and “psych”. E-mail addresses of clinical operators such as Ameos, Helios, and Sana were also checked and excluded. Finally, nonmedical e-mail addresses were excluded. In addition, missing information on medical specialization was obtained via internet research. Three automated electronic reminders were sent at 4-week intervals to respondents from the distribution list who had not yet participated. The data collection ended in August 2019. By this time, 34,027 of the 60,150 emails had been opened (56.6%), and 4177 had the survey link clicked (6,9%). In total, 1095 questionnaires were completed (1,8%). The response rate in relation to all recipients who followed the survey link and opened the survey was 26.2%. Among all the recipients who opened the e-mail 3.2%, and among all the e-mails sent, 1.8%.

### Data analysis

The data were analysed, and subgroups were formed using the online tools SurveyMonkey (Momentive Europe UC, Ireland) and Excel (Microsoft 365, Microsoft©, USA).

### Descriptive statistics

Since multiple responses were allowed for various questions, the number of responses given was related to the number of questionnaires answered. Due to this approach, a cumulative percentage above 100% was possible for questions with multiple answers. Descriptive statistical analysis was performed using nonparametric methods. In the subgroup analysis between general practitioners and specialists, the chi-square test was also applied. The significance level was set at *p* = 0.05.

## Results

### Study population

**Fig. 1:**
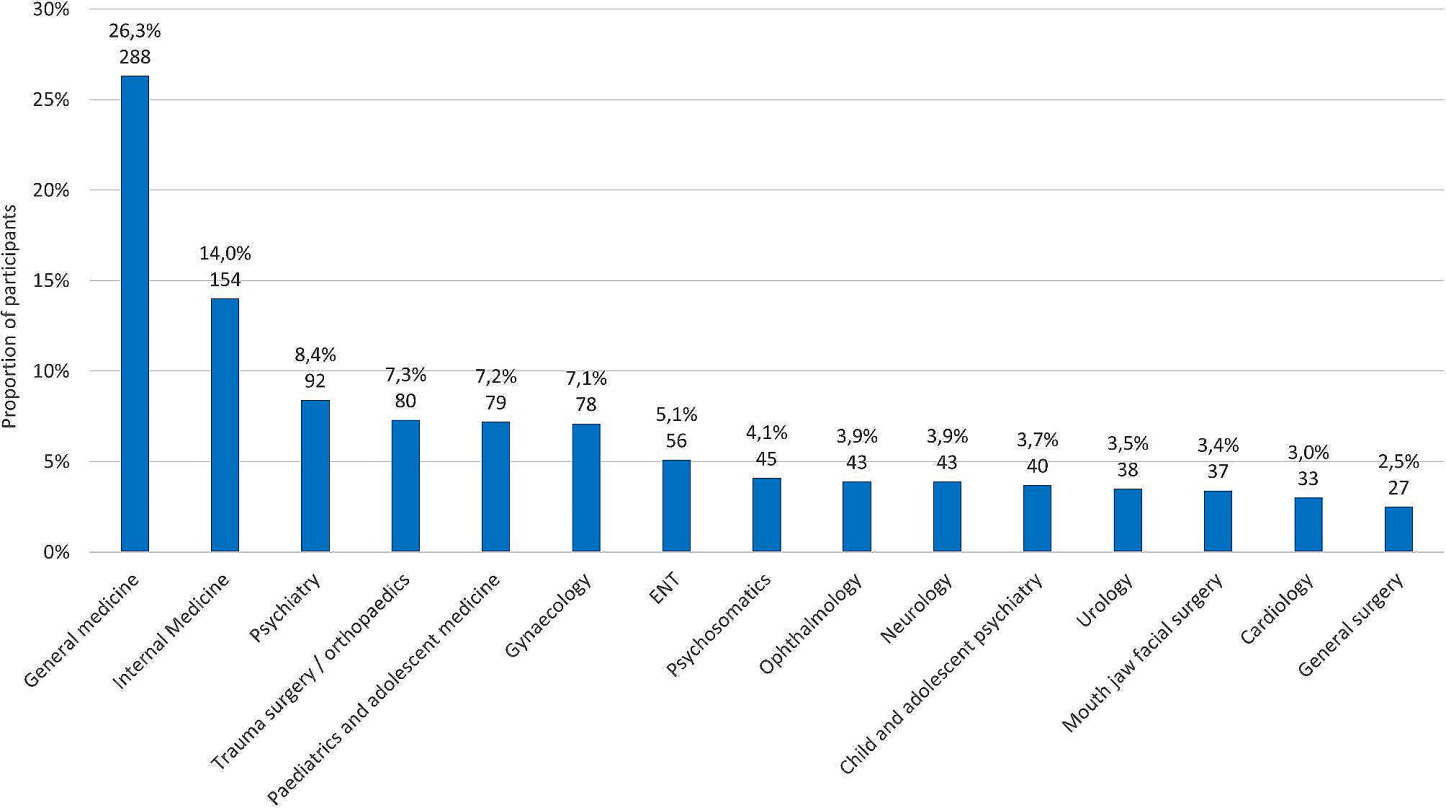
The 15 most frequently mentioned specialties (max. 2 answers). Note: The information is given in each case as a percentage and the absolute number (n)

A total of 1095 respondents were involved, 36.9% (405) were female, and the average age range of the physicians was 50 to 59 years old. A total of 45.2% (495) of the practitioners had worked in a hospital before setting up their practice, and on average, the physicians had been practising for 16 years. According to their own estimates, 38.9% (426) of the participants referred up to 10 patients per year to a university hospital, 32.6% (357) referred 11 to 30 patients per year, 14.1% (155) referred 31 to 50 patients and 14.3% (157) referred more than 50 patients per year to the nearest university hospital. Further details can be found in the supplemental material. Regarding specialty or clinical focus, respondents had the opportunity to choose 2 areas of focus. A list of the 15 most frequently mentioned specialties can be found in Fig. 1.

#### Current cooperation with university hospitals

The subjective overall impression of cooperation with the most frequently used department of the university hospital was rated as satisfactory by 70.6% of the responding physicians in private practice (very satisfied: 9.7%/106, “satisfied”: 25.7%/281; “rather satisfied”: 35.2%/385; “rather dissatisfied”: 18.4%/202; “dissatisfied”: 5.8%/63; “very dissatisfied”: 4.3%/47).

**Fig. 2:**
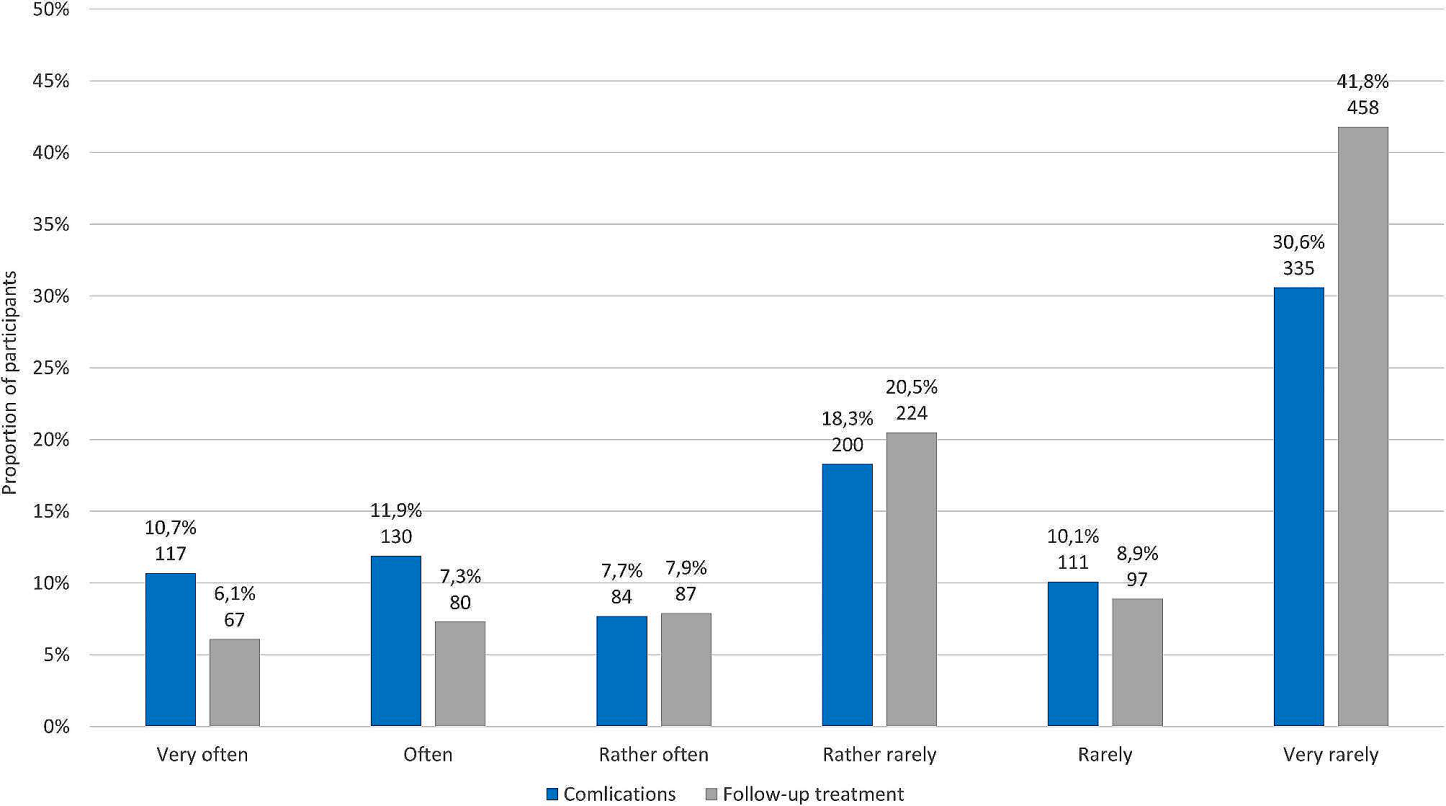
Responses regarding the frequency of notifications of complications occurring during inpatient stays and regarding planned follow-up treatments

The estimated waiting time for the inpatient discharge letter was 4 weeks (25%/75% quartiles: 2/8 weeks). The results regarding the frequency of notifications of complications occurring during inpatient stays and planned follow-up treatments are shown in Fig. 2.

**Fig. 3:**
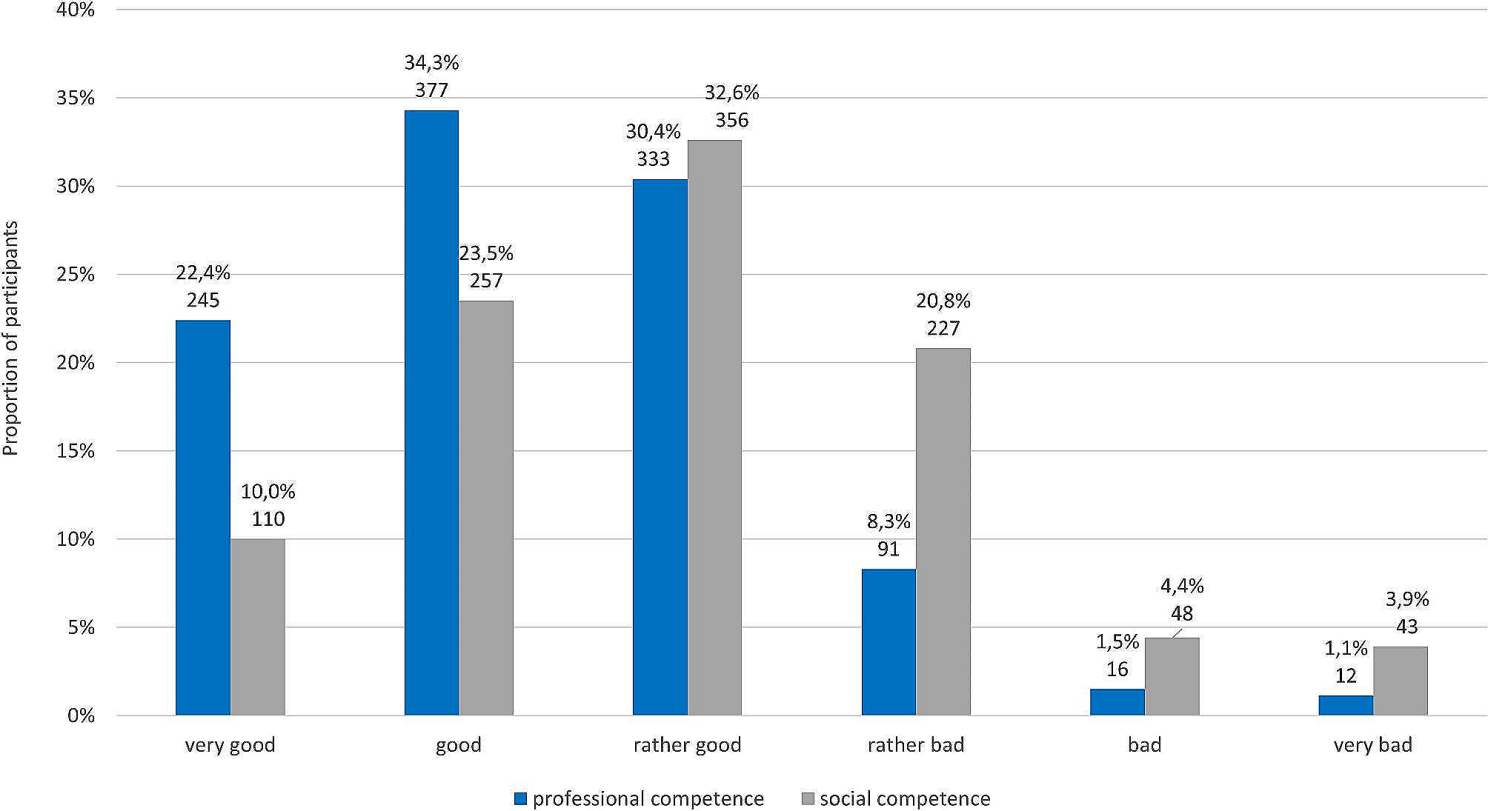
Perceptions of the professional and social competence of physicians in cooperating university departments Note: The information is given in each case as a percentage and the absolute number (n)

**Fig. 4:**
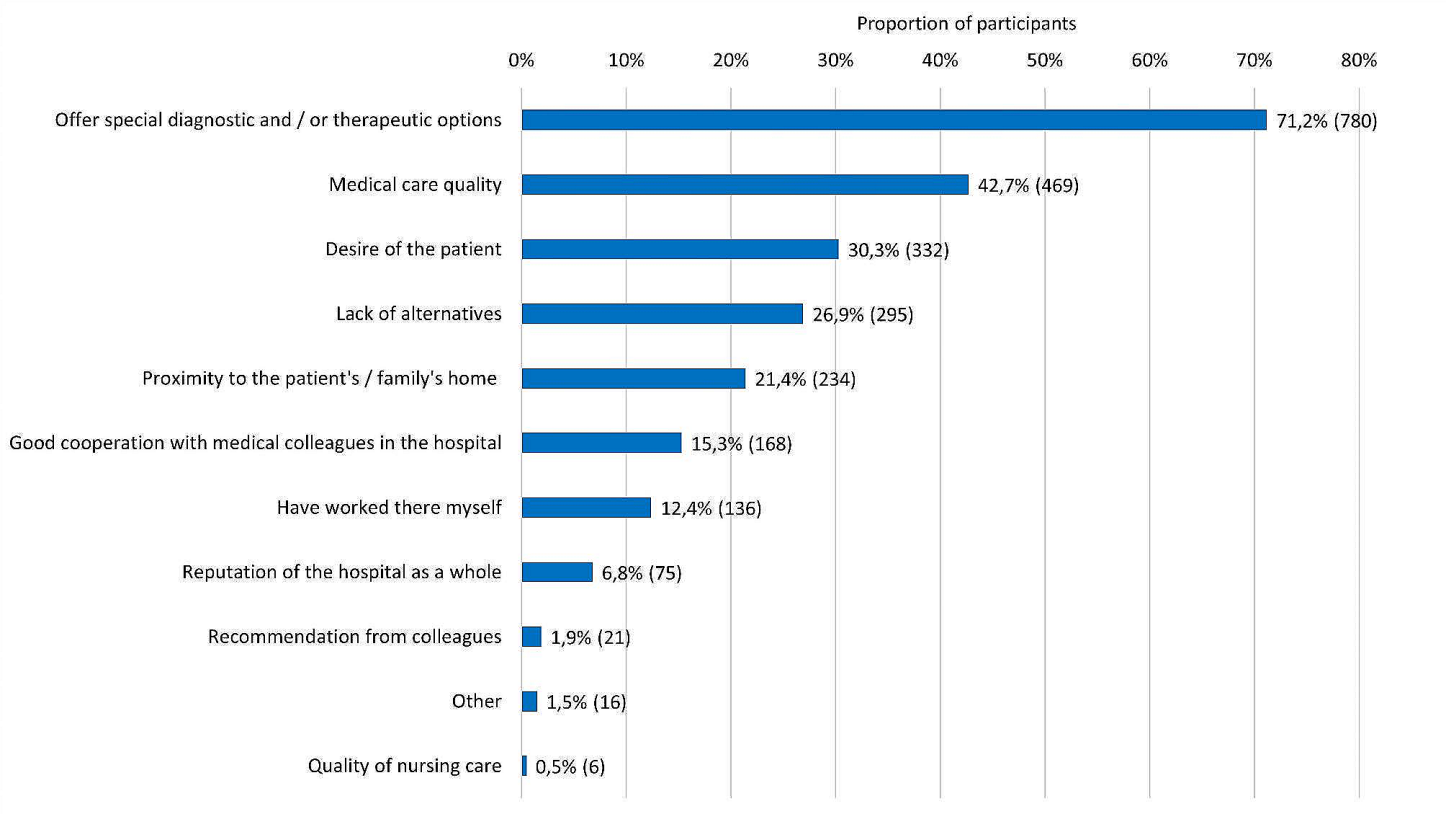
Reasons for referral to the university department (max. 3 answers) Note: The information is given in each case as a percentage and the absolute number (n)

The frequency of the unproblematic accessibility of a medical contact person who was competently informed about the patient and his or her condition was assessed heterogeneously by the respondents (“very often”: 9.3%/102, “often”: 13.6%/149; “rather often”: 21.9%/240; “rather rarely”: 26.9%/295; “rarely”: 11.4%/125; “very rarely”: 14.3%/157). The results of the perceptions of the professional and social competence of physicians in the cooperating university departments are displayed in Fig. 3. The reasons for referral to the university department are listed in Fig. 4.

#### Treatment quality

Responses to questions regarding the respondents’ satisfaction with subjective perceptions of treatment outcomes after discharge from the corresponding department showed the following distributions: very satisfied (10.0%/110), satisfied (38.8%/425), somewhat satisfied (38.4%/421), somewhat dissatisfied (9.0%/99), dissatisfied (1.3%/14), and very dissatisfied (0.9%/10).

The subjectively perceived frequency of complications occurring during hospitalization was described as very frequent (0.7%/8), frequent (3.2%/35), somewhat frequent (10.3%/113), somewhat rare (53.3%/584), rare (17.4%/190), or very rare (10.2%/112).

#### Development of cooperation and the potential for improvement

The statement “I consider the overall cooperation with the department of the university hospital to be in need of significant improvement” was answered in the affirmative by 68.3%/748 of the respondents (“totally agree”: 18.1%/198; “agree”: 24.2%/265; “rather agree”: 26.0%/285). Accordingly, 30.3%/332 disagreed with this statement (“rather disagree”, 18.7%/205; “disagree”, 8.3%/91; “ totally disagree”, 3.3%/36).

A total of 94.5%/1035 of the respondents agreed with the statement “An improvement in cooperation with the department of the university hospital would positively influence my bond with the university hospital” (absolute agreement: 54.6%/598, partial agreement: 39.9%/437). A total of 5.5%/60 of the respondents disagreed with the statement (rather disagree: 4.1%/45, disagree: 1.4%/15).

**Fig. 5:**
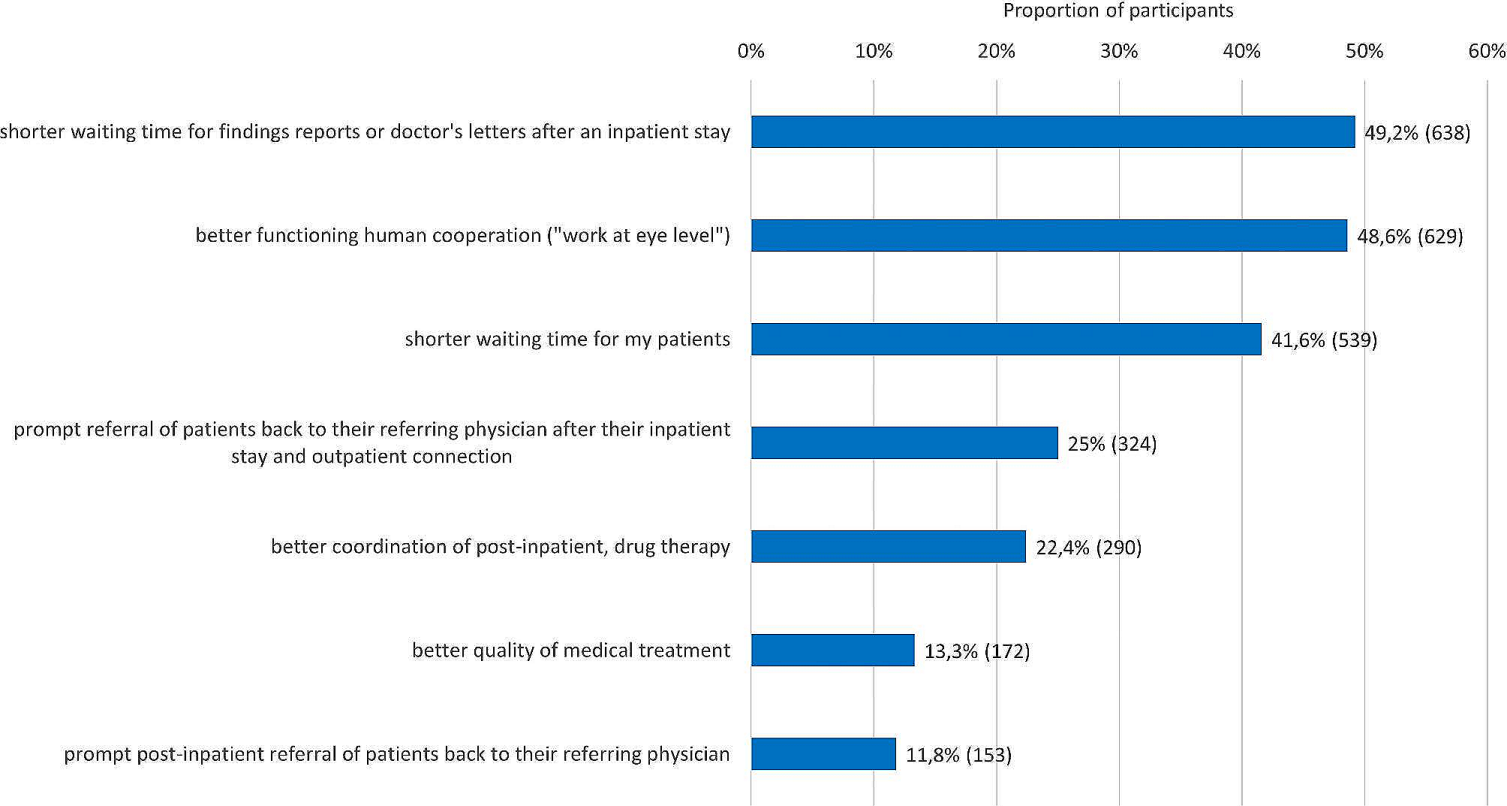
Responses to the question regarding what would benefit the collaboration with the relevant department of the university hospital: “if…” (max. 3 answers) Note: The information is given in each case as a percentage and the absolute number (n)

**Fig. 6:**
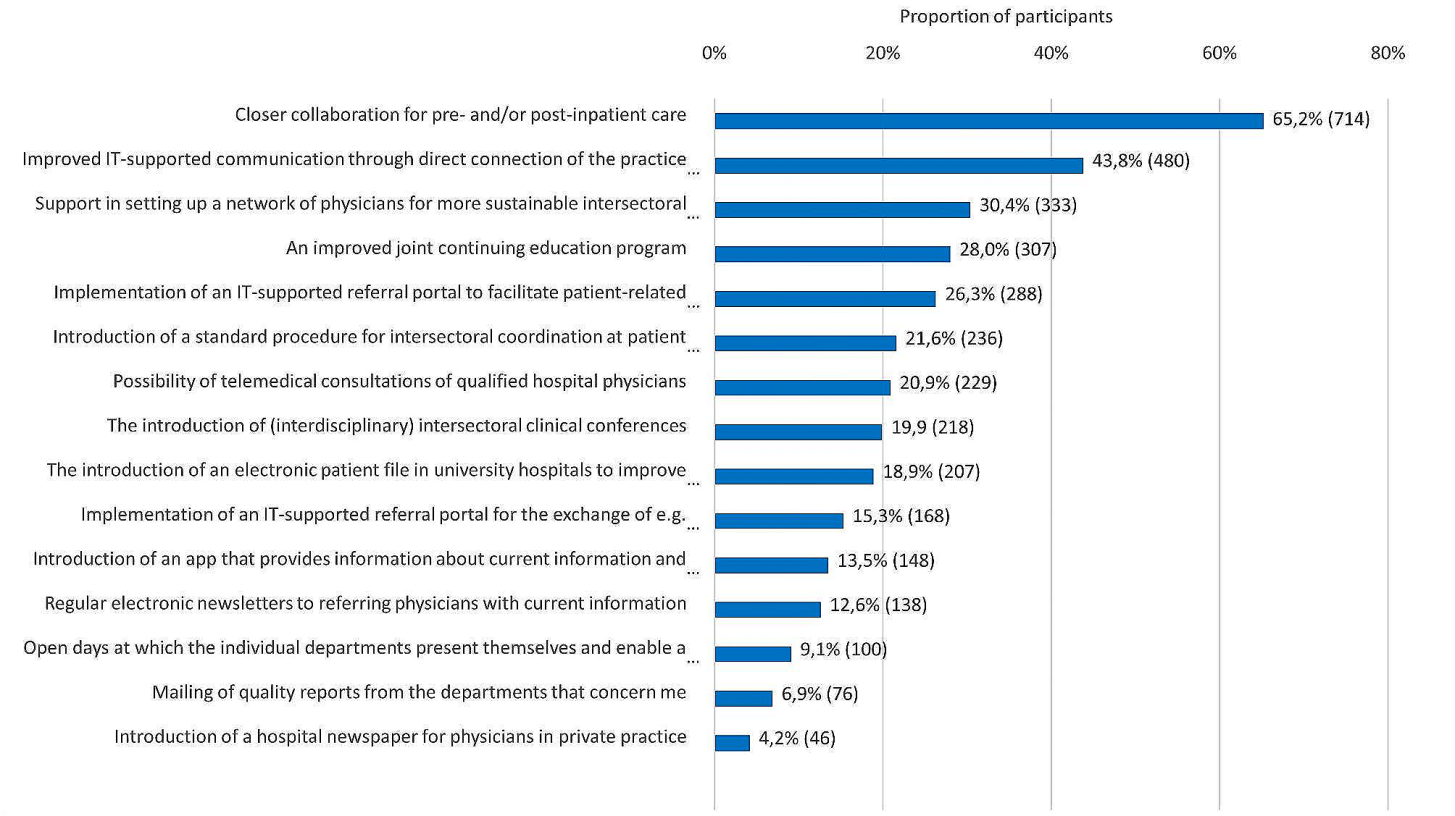
Answers to the question about concrete measures to improve cooperation (max. 5 answers) Note: The information is given in each case as a percentage and the absolute number (n)

The results regarding possible changes to improve collaboration with the relevant department of the university hospital are shown in Fig. 5. The relevance of concrete measures for improving cooperation is displayed in Fig. 6. Further details can be found in the tables and the text in the supplemental material.

### Subgroup analysis

To identify possible differences in the needs of general practitioners and specialists, these two subgroups were compared.

Both subgroups perceived the professional competence of their clinical colleagues in the same way (*p* = 0.9107), while the specialists rated the level of interpersonal competence better than the general practitioners did (68% rather than very positive compared to 55%; *p* < 0.0001). The specialists also stated that they were more likely to be able to reach a well-informed contact person by telephone (49% compared to 33%, *p* = 0.0003).

Approximately 7% of all general practitioners primarily referred patients to a university hospital, while referrals to a municipal hospital were much more common (62%). In contrast, approximately 27% of specialists primarily referred patients to a university hospital and 41% to a municipal hospital (*p* < 0.0001), with roughly the same distribution in terms of distance to the university hospital. Among the reasons for referral to a municipal hospital other than a university hospital, 62% of general practitioners (compared to 42% of specialists) reported better accessibility for patients and their relatives, 49% reported co-operation on an equal footing (compared to 39% of specialists), and 48% reported shorter waiting times for appointments (compared to 34% of specialists). The reasons for referral to university hospitals for general practitioners were special therapeutic and/or diagnostic options in 75% of cases (compared with 70% of specialists), the patient’s wishes in 35% of cases (compared with 26% of specialists) and a lack of alternatives in 40% (compared with 23% of specialists). The specialists used fewer answer options, and the answers were more varied. The most common reasons for referral to nonuniversity hospitals are accessibility for patients and relatives (42%), better human cooperation than with colleagues in university hospitals (39%) and shorter waiting times for patients (34%). As mentioned above, the primary reasons for referral to university hospitals for specialists are the range of special diagnostic and/or therapeutic options (70%) and the quality of medical care (39%). Both subgroups saw the potential for improvement in a very similar way, but the general practitioners saw significantly more potential for improved interpersonal collaboration (60% compared to 46% of the specialists) and better coordinated post inpatient treatment (44% compared to 17% of the specialists). Closer cooperation in both pre- and post inpatient care was also more important to them than to the specialists (72% compared to 50%).

Overall, the specialists in the survey generally had a slightly more positive view of the cooperating university hospital.

## Discussion

This is the first study to examine intersectoral collaboration from the referring physician’s perspective in a differentiated and independent manner in Germany.

### Current cooperation with university hospitals

A large proportion of the participating physicians in private practice were satisfied with the intersectoral collaboration overall, but they also saw clear potential for optimization. The most common reasons for referral to a university hospital were special diagnostic and therapeutic options and the quality of medical care. Here, there was a difference from nonuniversity hospitals, where referrals were made because of better accessibility for patients and relatives, faster appointments and better cooperation. This emphasizes the special medical role of university hospitals in Germany as maximum care providers but also shows potential for facilitating shorter waiting times and improved communication.

To improve the repeatedly mentioned limited accessibility of physicians in hospitals who are informed about patients, clear contact persons and reliable contact data are necessary; these data are made available to the referring physicians, e.g., online on the hospital homepage or an appropriately established referring physician portal, and are updated regularly. This approach has already been evaluated in a French study and showed good results [16]. Accessibility could be further improved, for example, by mobile service telephones. A quarter of the participants believed that there was relevant optimization potential in the improvement of these communication structures. In the future, a referral portal could also be used for cross-sector communication and the transmission of findings. However, according to only half of the participants, such a portal existed and was often described as “insufficient”, so there is relevant potential for optimization here. Alternatively, a standardized transmission of findings in the form of encrypted e-mails or via a secure portal should be examined.

In addition to the abovementioned results of the study by François et al., colleagues also described a high level of satisfaction among referring physicians, especially with the quality of treatment. Critical comments were also made about the discharge letter and the poor accessibility of the clinicians [16]. This shows that the intersectoral interface between physicians in private practice and hospitals also has similar strengths and weaknesses in other health care systems and that approaches to solutions that have been successfully introduced in other systems could therefore be examined and, if necessary, adopted. In general, however, it is important to further develop bidirectional communication since deficits in the transfer of information from the outpatient sector are likely to have comparable relevance for quality of care and patient safety.

Another relevant and similar aspect of communication is soft skills. The social competence of the clinicians was assessed by the majority of the respondents as good and thus relatively less positive compared to their professional competence. Accordingly, almost half of the participants stated that cooperation with the university department would benefit from better human interaction at the interpersonal level. In our survey, this previous lack of soft skills was one of the major reasons why patients were referred to a nonuniversity hospital. This potential for optimization can be realized through appropriate sensitization at the physician management level and through communication training. Likewise, the medical contact person in the hospital should also be selected based on his or her soft skills, and if necessary, specialist or senior physicians should be available for physicians in private practice. This could be achieved, for example, as part of the abovementioned introduction of mobile phones and telephone lists of clinical colleagues available to referring physicians. Ultimately, physicians in private practice are equal partners in patient treatment and not supplicants. Further measures to improve communication and collaboration relate to more intensive exchanges and dialogues between inpatient and outpatient physicians [19]. There are opportunities for regular joint training and/or “get-togethers”. This exchange already exists in part, but a significant expansion of this informal communication interface seems appropriate. In this way, improved intersectoral exchange and dialogue can lead to added value for patients and collaboration [23].

A central and frequently mentioned point of criticism by the respondents was the average 4-week waiting period for the discharge letter. Since this letter is often the only way for an outpatient caregiver to obtain a complete picture of the inpatient stay, measures taken and complications, as well as post inpatient drug therapy, this period is too long for this central aspect of interface communication. Correspondingly, almost half of the respondents stated that shortening the waiting time for the discharge letter or findings would lead to a relevant improvement in cooperation and thus also in the bond with the respective university hospital. In addition, since October 2017, according to the framework agreement of the Statutory Health Insurance, the National Association of Statutory Health Insurance Physicians, and the German Hospital Association, all hospitals are obligated to provide a physician’s letter at the time of discharge, including discharge findings, epicrisis, further procedures/recommendations, and medications [24].

Koné et al. conducted a survey of German GPs in 2018 to evaluate their satisfaction with cooperation with all other disciplines in the care of cancer patients, including outpatient oncology specialists, physicians in different hospitals, home care services and specialized palliative home care cancer care. A large part of the GPs interviewed in this survey perceived it as a problem that hospitals, for example, did not inform them about planned discharges, although this would have been considered important by the GPs. The GPs showed a fundamentally high level of satisfaction regarding cooperation with most other health care providers. They also generally rated them as competent as well. Both factors are strongly correlated with good communication and the timely exchange of information [22]. This perception of only general practitioners is in line with the perceptions of different disciplines of outpatient specialists expressed in our study. According to the majority of our respondents, relevant patient-related information, such as complications during the inpatient stay or information about follow-up treatment, was not provided or was provided but only to a limited extent. The framework agreement mentioned above also stipulates that the discharging physician is obliged to contact the person providing follow-up treatment in a timely manner before discharge, if necessary [24]. In many cases, this one contact would probably be sufficient to provide adequate information on patient-related information and information about follow-up treatment.

Against this background, the optimization of the waiting time for the discharge letter and the exchange of information represent central starting points within the framework of process optimization at the sector boundary and include an adjustment of prioritization as well as internal hospital processes. Missing information from an inpatient stay can lead to a relevant reduction in the quality of care in outpatient follow-up treatment. It has been repeatedly shown that communication deficiencies at this interface lead to a significantly increased risk of rehospitalization within a few weeks and incorrect medication adjustments [3–5]. Moreover, even single contact by clinicians increases referral satisfaction and improves patient safety [25].

The quality of discharge letters is a major factor for satisfaction itself. This phenomenon was explored in a study by Weetman et al., in which GPs and clinicians were asked about ‘successful’ and ‘unsuccessful’ discharge letters. Methods for ensuring the quality of letters can reduce patient risk at the time of hospital discharge [21]. One possibility to ensure this quality might be the implementation of standardized national discharge letters.

Overall, the model of the discharge letter sent by mail, as is often the case in Germany, must be discussed critically in modern times. There is an urgent need to integrate this intersectoral communication into the digital age. Various approaches can be implemented with varying degrees of effort:

- Sending an electronic physician’s letter via e-mail.
- The establishment of a patient-specific communication platform through which medical physicians can exchange information across sectors.
- The use of an electronic patient file.

### Treatment quality

The treatment outcomes were perceived very positively by the participants, and complications during the inpatient stay, which were assessed as rare, reflected satisfaction with the quality of medical treatment. This level of satisfaction regarding treatment outcomes and thus the professional quality of university hospitals coincides with the level of satisfaction with patient treatment in local surveys [15, 16, 26]. Here, we identified an essential aspect of referral retention that should be further developed by university hospitals despite overall satisfaction.

### Development of cooperation and the potential for improvement

With all the advantages and potentials of digitalization it makes little sense to use it in a scattergun approach since, as already examined, neither patient safety nor satisfaction will necessarily increase [27]. Additionally, with these options, differentiated access rights and the guarantee of appropriate data protection are mandatory prerequisites. In addition, the expansion of the telematic infrastructure and corresponding interfaces is important since increasing the digitization of communication can be realized only through the use of adequate information technology (IT) infrastructure in hospitals and clinical practices. These aspects correlate with our findings about what would benefit collaboration with university hospitals. Namely, shorter waiting times for discharge letters, improved communication—ideally via digital media—and better interpersonal cooperation would improve the intersectoral cooperation mentioned by the participants in this survey.

### Subgroup analysis

The subgroup analysis of general practitioners and specialists showed that specialists generally viewed the collaboration more positively than general practitioners did. This difference is presumably primarily due to differences in the reasons for referral. For specialists, the availability of special diagnostic and/or therapeutic options was by far the most important reason for referral to the nearest university hospital, while other frequently cited reasons for general practitioners included a lack of alternatives or the patient’s wishes. Specialists were also more likely to have previously been employed at university hospitals themselves. They also stated that they were able to reach a medical contact person by telephone significantly more often than were the general practitioners. However, both subgroups mentioned the potential for improvement in a very similar distribution or weighted them similarly. Although the idea of implementing a differentiated referral management system for GPs and specialists would be an interesting consideration due to their different needs and priorities, this idea would entail significant additional work with questionable added value. The implementation of the measures in the overall healthcare system would be a significant improvement, at least in Germany.

Due to the special situation in Germany compared to that in other countries, both with the clearly separated sectors of outpatient and inpatient health care and with an IT infrastructure in the health care system that is still in need of significant development, these recommendations cannot necessarily be transferred to other countries.

### Limitations

A commercially purchased email distribution list was used, which originally consisted of 103,000 email addresses. During quality control, the distribution list was reduced to 60,150 email addresses. In addition, of these remaining email addresses, many were presumably no longer valid or active, as only 34,027 were opened. The survey link was clicked on only 4177 of these opened emails. It is unclear whether the low number of activated links was due to a lack of willingness to participate in the survey or due to deficiencies in the distribution list. Another aspect would be an initial screening and filtering of emails by nurses or physician’s assistants rather than the physician himself, so that the physician has no knowledge of the survey. Overall, the response rate must be discussed critically because the 1095 responses from human medicine physicians in private practice can be seen only in relation to the 60,150 physicians who were contacted; however, as described here, only a limited proportion of the responses were actually reached. Even if the results presented are not necessarily representative, we consider the potential for improvement found here to be relevant since more than 1,000 physicians in private practice presented their perspective in detail.

Some responses reflected survey fatigue or frustration regarding descriptive intersectoral research that does not achieve direct change in the system. Another limiting factor regarding willingness to participate is time and economic pressures in the outpatient setting [28]. Furthermore, it could be shown that an insufficient perception of oneself as an equal partner and the lack of influence of the research process also negatively influence the cooperation of physicians in private practice [28].

In addition, it must be taken into account that only the perspective and subjective experiences of physicians in private practice were surveyed. However, intersectoral collaboration is not a one-way street. The perspective of the hospitals is just as relevant for the overall picture and should be collected in the context of further studies. One study has shown that there is also room for improvement in the area of communication, e.g., availability by telephone and documents for referrals and rereferrals from the perspective of inpatient physicians [29].

Another limitation is the restriction of the survey to university hospitals. This restriction was due to easier comparability since university hospitals generally offer comparable conditions in terms of size, number of beds and departments, and equipment. In addition, most of the physicians in private practice had only one university hospital in their region, which made it possible for the respondents to clearly assign their own experiences. It should be noted that the optimization potentials presented here inevitably do not apply to all departments of a university hospital or to nonuniversity hospitals.

## Conclusion

The following aspects should be critically examined in university hospitals—and in nonuniversity hospitals—and, if necessary, established or further developed:

- Clear presentation of contact persons online, with contact options and times.
- Improved accessibility by telephone.
  - special (mobile) service numbers for patients in private practice.
  - coordinating secretariat (if necessary, per department).
- Referral portals that enable the transmission of physicians’ letters and findings both from referrers (for admission) and from hospitals (for discharges).
- Regular dialogue in the form of continuing education and/or “get-togethers”.
- Timely discharge letters through the optimization of internal hospital processes.
- Further development of cross-sector communication channels with a particular focus on digital formats.
- Further development of electronic documentation of treatment data and IT-supported communication.
- Regular surveys of referring physicians and communication of the results and resulting measures.

### Electronic supplementary material

Below is the link to the electronic supplementary material.

Supplementary Material 1

Supplementary Material 2

Supplementary Material 3

Supplementary Material 4

Supplementary Material 5

Supplementary Material 6

Supplementary Material 7

## Data Availability

All the data generated or analysed during this study are included in the supplementary data file “Raw data”.
