# Supplementary figures and images for: Intersectoral cooperation between university hospitals and physicians in private practice in Germany– where the potential for optimization lies
Source: BMC Health Serv Res. 2024 Apr 22;24:497. doi: 10.1186/s12913-024-10963-8 (PMC11034040; doi:10.1186/s12913-024-10963-8)

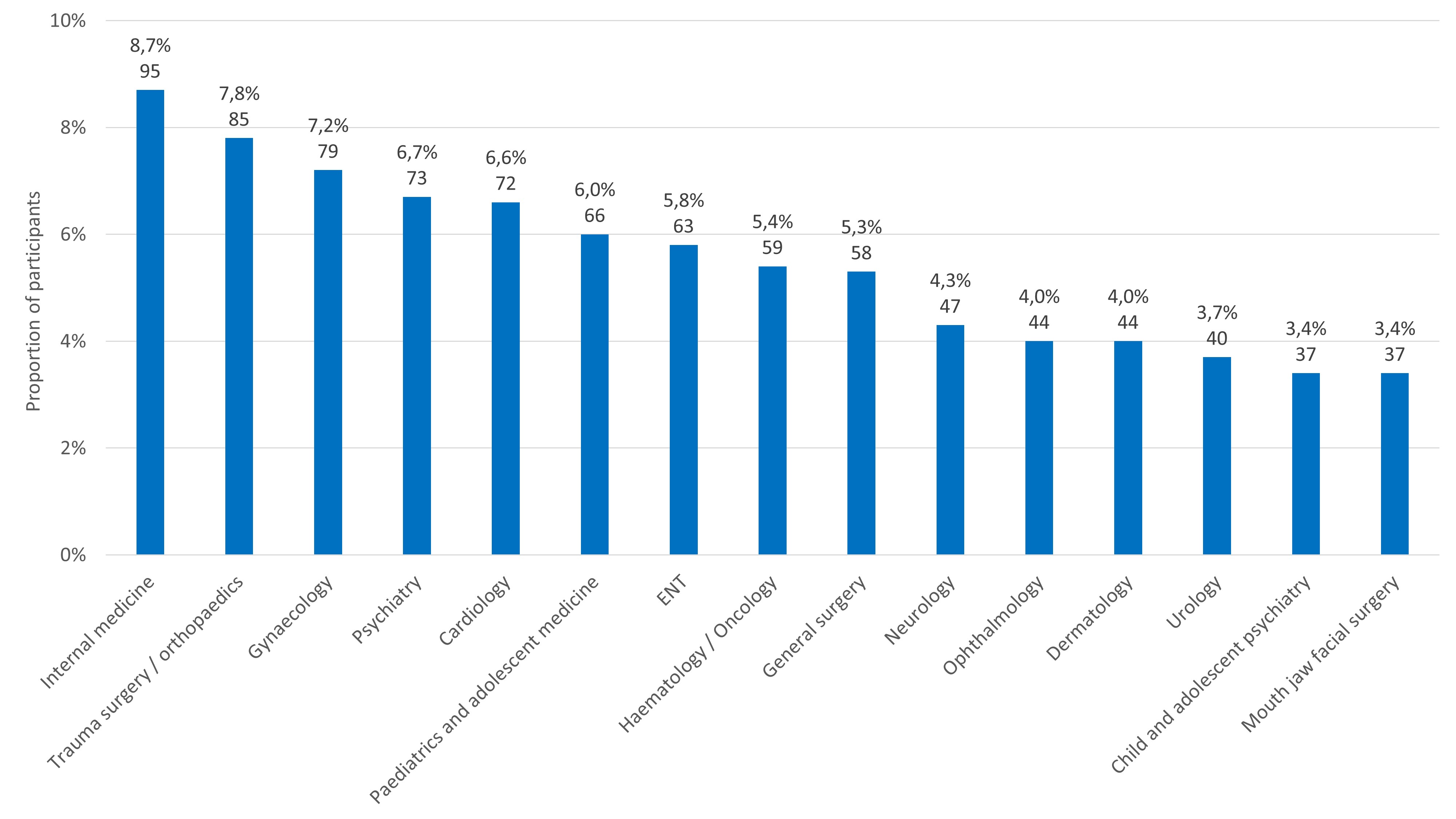

Supplement: Supplementary file 2 — Supplementary Material 2 [file 12913_2024_10963_MOESM2_ESM.jpg]

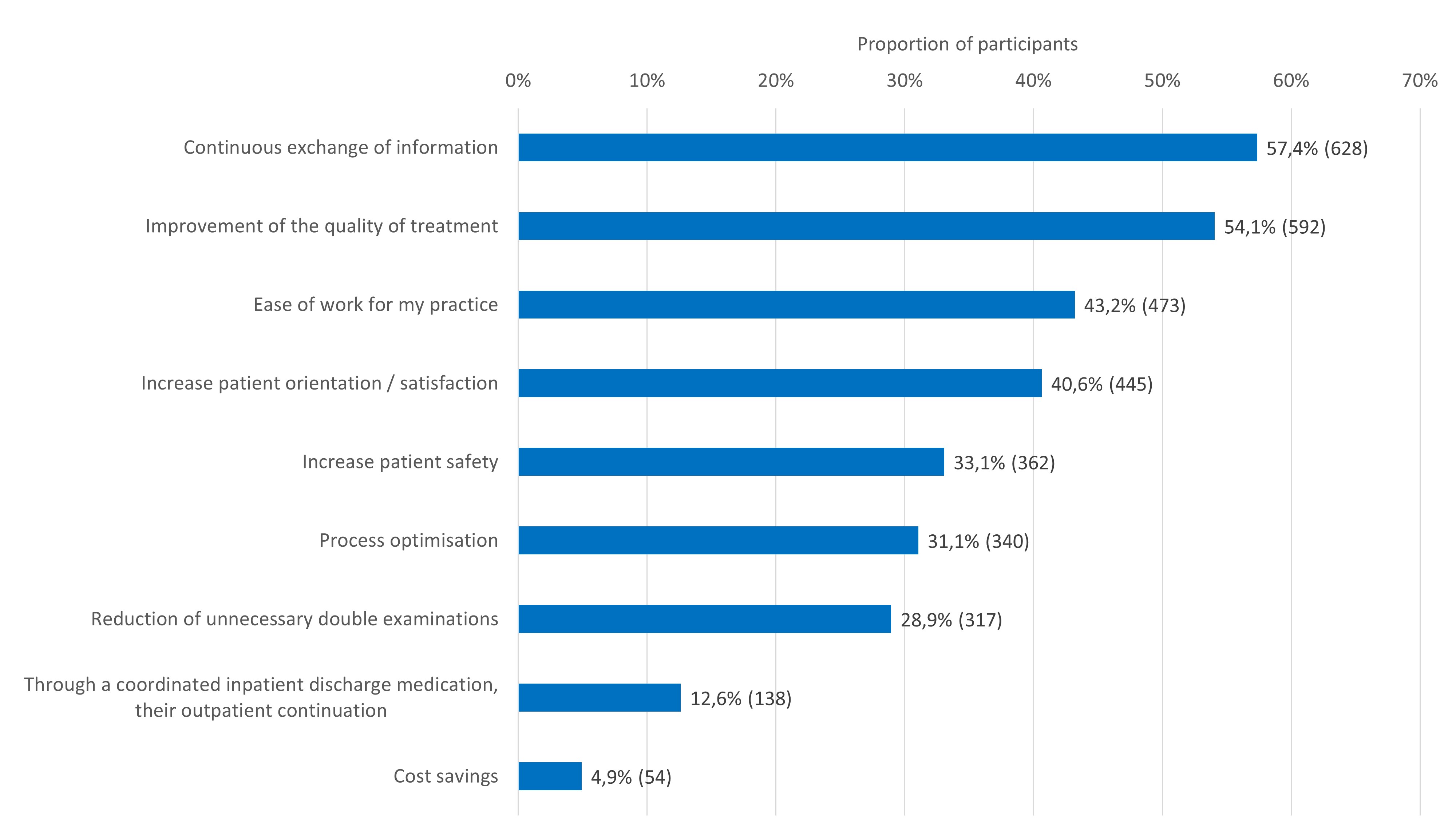

Supplement: Supplementary file 3 — Supplementary Material 3 [file 12913_2024_10963_MOESM3_ESM.jpg]

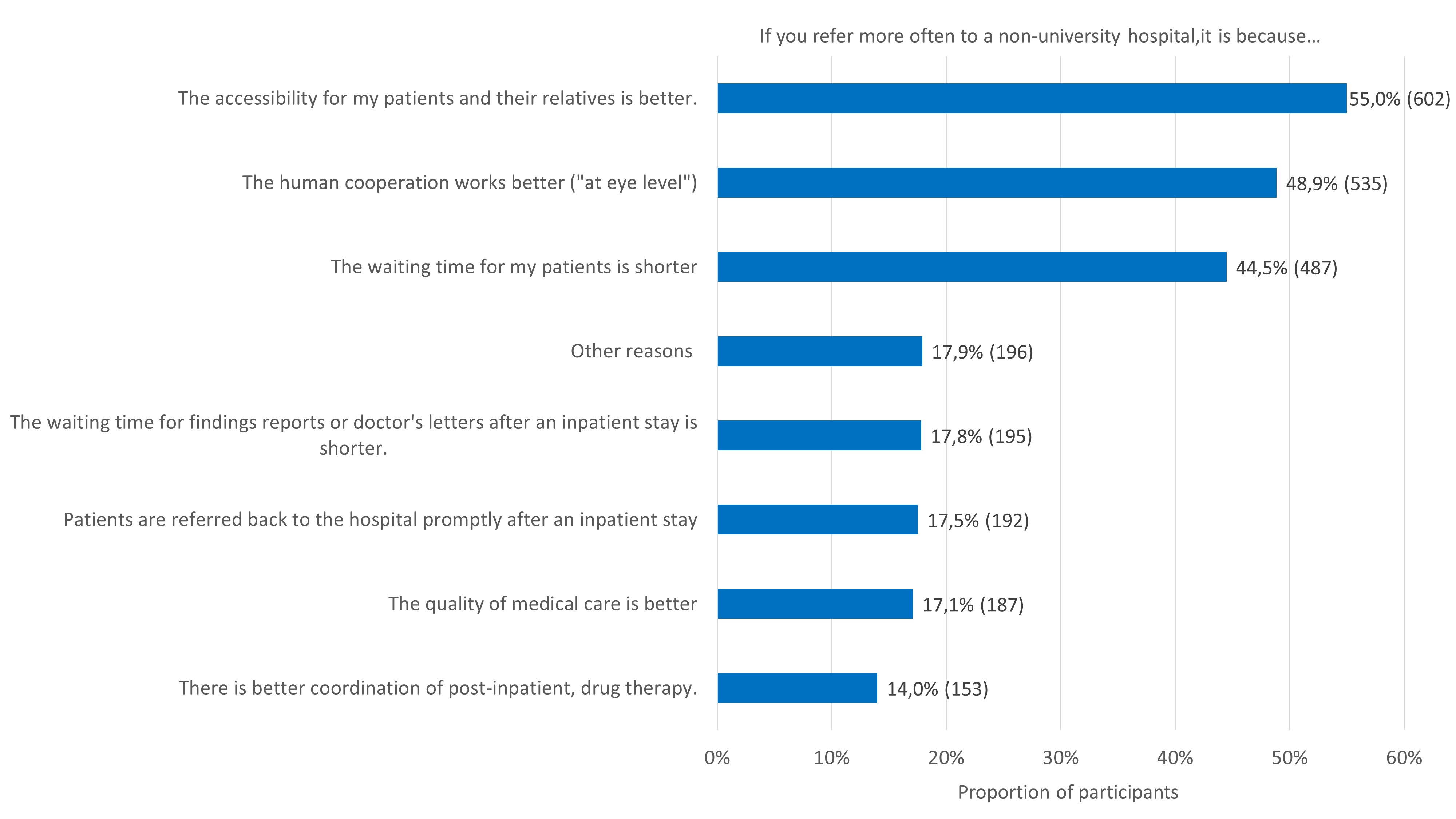

Supplement: Supplementary file 4 — Supplementary Material 4 [file 12913_2024_10963_MOESM4_ESM.jpg]
