# Supplementary material for: Intersectoral cooperation between university hospitals and physicians in private practice in Germany– where the potential for optimization lies
Source: BMC Health Serv Res. 2024 Apr 22;24:497. doi: 10.1186/s12913-024-10963-8 (PMC11034040; doi:10.1186/s12913-024-10963-8)
Supplement: Supplementary file 5 — Supplementary Material 5 [file 12913_2024_10963_MOESM5_ESM.docx]

**Table S1**

| **General information (n=**1095; **%/ n)** | | | |
| --- | --- | --- | --- |
| Average time working in hospital before establishment (In years) | | | 9 |
| Distance to the nearest university hospital | | | |
| - Up to 5 km | | | 19,9% (218) |
| - 6 – 20 km | | | 29,7% (325) |
| - 21 – 50 km | | | 25,0% (274) |
| - Over 50 km | | | 25,4% (278) |
| Type of establishment | | |  |
| - Practice (co-)owner | | | 80,7% (884) |
| - Part of a joint practice | | | 9,2% (101) |
| - Part of a group practice | | | 27,2% (298) |
| - Employed physician | | | 2,2% (25) |
| - Employed physician in an MVZ | | | 2,1% (23) |
| **Current cooperation with university hospitals (%/n)** | | | |
| Waiting time for appointment for elective admission (in days; median; quartiles) | 14  (10/30) | | |
| Frequency of switching discharge medication to generic drugs, excluding switching | | | |
| - Very often | 12,2% (134) | | |
| - Frequently | 14,2% (155) | | |
| - Rather frequently | 19,2% (210) | | |
| - Rather rarely | 28,0% (307) | | |
| - Rarely | 6,4% (70) | | |
| - Very rarely | 9,0% (99) | | |
| Frequency of discontinuation of inpatient medication without substitution | | | |
| - Very often | 3,3% (36) | | |
| - Frequently | 6,3% (69) | | |
| - Rather frequently | 13,0% (142) | | |
| - Rather rarely | 37,1% (406) | | |
| - Rarely | 11,1% (121) | | |
| - Very rarely | 20,5% (224) | | |
| The university hospital's continuing education programme is attractive and balanced in terms of the frequency and quality of the events. | | | |
| - Totaly agree | 6,4% (76) | | |
| - Agree | 28,1% (308) | | |
| - Likely to agree | 28,3% (310) | | |
| - Likely to disagree | 15,5% (170) | | |
| - Disagree | 7,0% (77) | | |
| - Does not apply at all | 5,8% (63) | | |
| **Quality of treatment (%/n)** | | | |
| Would you recommend the department to friends or acquaintances if it were indicated? | | | |
| - Yes, definitely | | 25,2% (276) | |
| - Yes, probably | | 53,9% (590) | |
| - No, rather not | | 17,4% (190) | |
| - No, definitely not | | 3,6% (39) | |
| **Development of cooperation and potential for improvement (%/n)** | | | |
| Development of cooperation in the past 10 years | | | |
| - Significantly improved | | 6,3% (69) | |
| - Improved | | 27,3% (299) | |
| - Remained the same | | 45,5% (498) | |
| - Deteriorated | | 18,1% (199) | |
| - Significantly worsened | | 2,7% (30) | |
| **Comparison of the university hospital with the non-university hospital (%/n)** | | | |
| Assessment of the cooperation with the university hospital as a whole compared to that with the non-university hospital, with which cooperation is most frequent? | | | |
| - Much better | | 7,6% (83) | |
| - Better | | 9,3% (102) | |
| - Rather better | | 21,7% (238) | |
| - Rather worse | | 42,8% (469) | |
| - Worse | | 9,7% (106) | |
| - Much worse | | 8,9% (97) | |
| Frequency of patient admission to university hospital compared to non-university hospital? | | | |
| - Much more often | | 7,9% (87) | |
| - More often | | 8,4% (92) | |
| - Rather more often | | 11,8% (129) | |
| - Rather less often | | 37,6% (412) | |
| - Less often | | 14,0% (153) | |
| - Much less often | | 20,3% (222) | |
| Where do you most often refer your patients for an inpatient stay? | | | |
| - Municipal hospital | | 48,4% (530) | |
| - Private hospital | | 12,0% (131) | |
| - Denominated hospital | | 22,0% (241) | |
| - University hospital | | 17,6% (193) | |
| Have you ever been questioned by the specialist department or otherwise by staff of the resident university hospital regarding your satisfaction and/or an improvement of the cooperation? | | | |
| - Yes | | 31,9% (349) | |
| - No | | 68,1% (746) | |
| If yes, by whom? | | | |
| - Head physician of the department | | 45,0% (108) | |
| - Senior physician of the department | | 24,2% (58) | |
| - Quality management staff | | 12,1% (29) | |
| - Management/executive board | | 7,5% (18) | |
| - Staff oft he referral management | | 4,2% (10) | |
| - Others | | 7,1% (17) | |
| Do you have the impression that something has changed as a result of your answers in such a survey? | | | |
| - Yes | | 41,0% (143) | |
| - No | | 51,0% (178) | |
