# Supplementary material for: Intersectoral cooperation between university hospitals and physicians in private practice in Germany– where the potential for optimization lies
Source: BMC Health Serv Res. 2024 Apr 22;24:497. doi: 10.1186/s12913-024-10963-8 (PMC11034040; doi:10.1186/s12913-024-10963-8)
Supplement: Supplementary file 6 — Supplementary Material 6 [file 12913_2024_10963_MOESM6_ESM.pdf]

Study "Current status of intersectoral cooperation - A survey on the perspective of physicians in private practice in Germany"

Please help us to assess the current status of your cooperation with university departments or university hospitals, to identify weaknesses in the interaction with you as a referrer and to develop future possibilities of cross-sectoral cooperation in order to achieve sustainable further development for the benefit of our patients.

Dear Colleague,

Cross-sectoral cooperation between physicians in private practice, such as yourself, and university hospitals has a significant influence on the quality of treatment and patient safety. In everyday clinical practice, there is always considerable potential for optimisation. So far, however, there is a lack of well-founded information regarding the perception of colleagues in private practice regarding this cooperation. For this reason, we are writing to you today and asking for your opinion. We - a health services research group of the University Medical Center Göttingen - want to present the current state of cooperation at this important interface, identify possible potential for improvement and develop appropriate measures. Please take part in the anonymised survey via the link below so that we can jointly further develop crosssector interaction in the interests of our patients and ourselves. Your Association of Statutory Health Insurance Physicians was informed in advance and approves participation in this survey.

If you have any questions or comments, please do not hesitate to contact us by e-mail at timpeter.epperlein@stud.uni-goettingen.de or by mobile at +49 176 83450158.

With friendly, collegial greetings

Tim Epperlein PD Dr Reiner M. Wäschle, MaHM Prof. Dr Michael Quintel

Editing note

When answering the questions, please consider your own experience of working with the specialist department of the university hospital with which you mainly cooperate. If you have several university hospitals in your catchment area, please refer to the university hospital with which you cooperate most frequently. The same applies to the cooperation with non-university hospitals at the end of the questionnaire. Non-university hospitals (non-UK) are all municipal, denominational, or private hospitals, including teaching hospitals, which are not themselves university hospitals. Please also answer this question based on the non-UK with which you cooperate most frequently.

Current cooperation with university hospitals

1. On average, how long do your elective patients have to wait for an appointment for inpatient admission at the university hospital? (In days)

2. If complications arise in one of your patients during their inpatient stay or if they die, how regularly are you informed by the hospital doctors treating them?

   Very often - often - rather often - rather rarely - rarely - very rarely/ never - no answer

3. How often are you informed and/or involved regarding follow-up treatment (selection of a post-inpatient rehabilitation/nursing facility)?

   Very often - often - rather often - rather rarely - rarely - very rarely/ never - no answer

4. On average, how long do you wait for the final, comprehensive doctor's letter regarding inpatient care after your patient has been discharged from the university hospital? (In weeks)

5. How often can you easily reach a competent medical contact by phone? Very often - often -

   rather often - rather rarely - rarely - very rarely - no answer

6. How do you rate the professional competence of the doctors in the respective specialist department of the university hospital?

   Very good - good - rather good - rather poor - poor - very poor

7. How do you rate the human competence of the doctors in the respective specialist department of the university hospital?

   Very good - good - rather good - rather poor - poor - very poor

8. How often do you change the discharge medication for your patients after inpatient treatment in the respective specialist department of the university hospital?

   Very often - often - rather often - rather rarely - rarely - very rarely/ never – none

9. How often do you discontinue inpatient prescribed medication without replacement?

   Very often - often - rather often - rather rarely - rarely - very rarely/ never - none

10. Why do you refer patients to the cooperating specialist department of the university hospital? (Multiple answers max. 3 answers)

    a. Wish of the patient

    b. Proximity to the patient's/ relatives' place of residence

    c. Missing alternative

    d. Quality of medical care

    e. Offer special diagnostic and/or therapeutic options

    f. Quality of nursing care

    g. Good cooperation with the medical colleagues in the hospital

    h. Recommendation from colleagues

    i. Worked there myself

    j. Reputation of the hospital as a whole

    k. Other

11. How satisfied are you overall regarding the cooperation with the specialist department of the university hospital?

    Very satisfied - satisfied - rather satisfied - rather dissatisfied - dissatisfied – very dissatisfied

Text box: The term "referral management" used in the following refers to an organisational unit of a hospital that coordinates and develops the cooperation with referrers with regard to common patients and, if necessary, obtains feedback.

12. How would you rate the following statement in relation to the cooperation with the university hospital as a whole?

    a. The university hospital has a superordinate, central referral management system.

    b. The university hospital's referral management is mature and requires only minimal adjustments.

c. The continuing education programme offered by the University Hospital is attractive and balanced in terms of the frequency and quality of the events.

d. The IT-supported transmission of findings between me and the university hospital runs smoothly and helps to avoid unnecessary duplicate examinations.

e. The University Hospital has a functioning, digital referral portal that allows you as a referrer to access case-specific (examination results, doctors' letters, etc.) and organisational (e.g. display of available beds) information in a user-friendly way.

Strongly agree - Agree - Somewhat agree - Somewhat disagree - Disagree - Disagree at all

Quality of treatment

13. How often do unexpected complications occur post-inpatient in your patients?

    Very frequently - frequently - rather frequently - rather rarely - rarely - very rarely

14. How satisfied are you with the treatment results of your discharged patients after treatment in the respective specialist department of the university hospital?

    Very satisfied - satisfied - rather satisfied - rather dissatisfied - dissatisfied – very dissatisfied

15. Would you recommend the specialist department of the university hospital to friends or acquaintances with the appropriate indication?

    Yes, definitely - Yes, probably - No, rather not - No, definitely not

Development of cooperation and potential for improvement

16. How do you rate the following statement: "You consider the cooperation with the specialist department of the university hospital to be in need of significant improvement overall"?

    Strongly agree - Agree - Somewhat agree - Somewhat disagree - Disagree – disagree at all

17. In the past 10 years, the cooperation with the specialist department of the university hospital has developed as follows:

    Significantly improved - improved - remained the same - worsened - significantly worsened

18. Cooperation with the relevant specialist department of the university hospital would benefit significantly if (multiple answer, maximum 3 answers)

    a. the waiting time for my patients would be shorter.

    b. the waiting time for findings reports or doctors' letters after an inpatient stay would be shorter.

    c. the quality of medical treatment would be better.

    d. human cooperation would work better ("at eye level").

e. a better coordination of post-inpatient, drug therapy would take place.

f. a post-inpatient referral back would be made in a timely manner.

19. I consider the following measures of a specialist department or the university hospital to be important with regard to better cooperation (multiple answer, maximum 5 answers)

a. the introduction of (interdisciplinary) intersectoral clinical conferences.

b. an improved training offer (general practitioners + hospital doctors)

c. closer cooperation in pre- and/or post-inpatient care

d. improved IT-supported communication through direct connection of the practice IT-Systems with the hospital information system

e. Possibility of telemedical consultations from qualified hospital doctors

f. Introduction of an electronic patient file in the university hospitals for improved information exchange

g. Introduction of a standard procedure for intersectoral coordination at patient discharge

h. Introduction of a mobile application ("app") that allows you to find out about current information and contacts at the hospital.

i. Implementation of an IT-supported referral portal to facilitate patient-related communication with referrers

j. Implementation of an IT-supported referral portal that enables an exchange of findings and allows me to call up free bed capacities and book them, if necessary, and supports coordination and information regarding the discharge date.

k. Support for the establishment of a doctors' network with regular meetings for the sustainable networking of doctors in outpatient and inpatient health care ("Ärztestammtisch" or "Get together").

l. Regular electronic newsletters to the referrers with current information

m. Open days where the individual departments present themselves and enable dialogue.

n. Dispatched of the quality reports the me concerning me departments of the university hospital

o. Introduction of a clinic newspaper for practising doctors

p. Free text

20. What do you see as the greatest gains in improving cooperation resulting from the measures marked above? (Multiple answers, maximum 3 answers)

a. Improving the quality of treatment

b. Continuous exchange of information (professional vs patient-specific)

c. Increase patient orientation/satisfaction

d. Increase patient safety

e. Making work easier for my practice

f. Reduction of unnecessary duplicate examinations

g. Through an agreed, inpatient discharge medication, their outpatient continuation

h. Cost saving

i. Process optimisation

21. How would you rate the following statement:

"An improvement in the cooperation with the specialist department of the university hospital would positively influence my bond with the university hospital."?

I absolutely agree - I partly agree - I rather disagree - I absolutely disagree

Comparison university hospital - non-university hospital

Please rate the following statements comparing the cooperation with your university hospital and the non-university hospital with which you cooperate most often.

22. Overall, how would you rate the cooperation with the university hospital compared to the non-university hospital with which you cooperate most often?

Significantly better - better - rather better - rather worse - worse - significantly worse

23. How often do you refer patients to the university hospital compared to the non-university hospital?

Much less often - less often - rather less often - rather more often - more often – much more often

24. If you refer more often to a non-university hospital, it is because (multiple answer, maximum 3 answers)

a. the waiting time for my patients is shorter.

b. the waiting time for findings reports or doctor's letters after an inpatient stay is shorter.

c. the accessibility for my patients or their relatives is better.

d. the quality of medical treatment is better.

e. human cooperation works better ("at eye level").

f. a better coordination of post-inpatient, drug therapy takes place.

g. a post-inpatient referral back of patients takes place promptly.

h. More reasons

25. Where do you most often refer your patients for an inpatient stay?

a. Municipal hospital

b. Private hospital

c. Denominational hospital

d. University Hospital

Questions about the person

26. Gender

27. Age (in decades)

28. How long did you work in the hospital before (incl. training period)?

29. Were you (temporarily) employed at a university hospital prior to your establishment?

30. What is your clinical focus?

General medicine	Oral and maxillofacial surgery
General surgery	Nephrology
Anaesthesia	Neurosurgery
Ophthalmology	Neurology
Dermatology	Psychiatry
Gastroenterology	Radiology
Gynaecology	Rheumatology
Otorhinolaryngology	Sports Medicine
Internal medicine	Thoracic cardiovascular surgery
Cardiology	Trauma surgery
Laboratory Medicine	Urology
Microbiology/ Hygiene/ Infectiology	Dentistry

31. Are you a general practitioner or a specialist?

32. How many patients do you refer to a specialist department at the university hospital per quarter?

a. < 6

b. 6-10

c. 11 - 15

d. 16-25

e. >25

f. So rare that I don't have a decided opinion

33. How far is your practice from the university hospital?

    a. 0-5 km;

    b. 6-20 km;

    c. 21-50 km;

    d. > 50 km

34. What is the postcode of the university hospital with which you primarily work?

35. How long have you been working in the current practice?

36. Is this your first practice?

37. How long have you been in practice in total?

38. Are you or is it: (multiple answer)

    a. Owner

    b. Employee

    c. A group practice

    d. A community of practice

39. Have you already been surveyed by the department or university hospital regarding your satisfaction? #

    Yes/No

40. Have you already been approached by employees of the resident university hospital regarding improving cooperation?

    Yes/No

41. If yes, by whom:

    a. Management/ Board of Directors

    b. Head of Department

    c. Senior physician of the department

    d. Other

42. Do you have the impression that something has changed because of your answers in such a survey?

    Yes/No

Comments/Note

Please provide us with further information (in particular possible improvements) for your cooperation with the local university hospital
