# Supplementary material for: Intersectoral cooperation between university hospitals and physicians in private practice in Germany– where the potential for optimization lies
Source: BMC Health Serv Res. 2024 Apr 22;24:497. doi: 10.1186/s12913-024-10963-8 (PMC11034040; doi:10.1186/s12913-024-10963-8)
Supplement: Supplementary file 7 — Supplementary Material 7 [file 12913_2024_10963_MOESM7_ESM.docx]

**Supplement**

**Table S1:** General information about respondents, overview of survey results on current collaboration, and results comparing university hospitals and non-university hospitals.

The 15 departments to which respondents most frequently referred are shown in Figure 1

**Figure S1:** The 15 most frequently named departments to which respondents referred

Note: The information is given in each case as a percentage and the absolute number (n).

The statement that the university hospital has a superordinate, central referral management, which coordinates the cooperation with referrers as a superordinate organizational unit of a hospital, applies according to 42.5% of the respondents ("Applies completely": 5.9%/65, "Applies": 20.7%/227, "Applies rather": 15.9%/174, "Applies rather not": 19.2%/210, "Does not apply": 14.8%/162, "Does not apply at all": 13.2%/145). The statement "The University Hospital's referral management is mature and requires only minimal adjustments." was agreed to by 30.8% ("Strongly agree": 2.6%/28, "Agree": 11.9%/130, "Somewhat agree": 16.3%/178, "Somewhat disagree": 22.0%/241, "Disagree": 15.9%/174, "Disagree at all": 18.8%/206).

"The IT-supported transmission of findings between me and the university hospital runs smoothly and helps to avoid unnecessary duplicate examinations" was predominantly rated as not true ("Strongly agree": 1.6%/17, "Agree": 4.7%/51, "Somewhat agree": 7.7%/84, "Somewhat disagree": 13.0%/142, "Disagree": 18.2%/199, "Disagree at all":40.2%/440).

The same applies to the statement "The university hospital has a functioning, digital referral portal that gives you as a referrer access to case-specific (examination results, doctor's letters, etc.) and organizational (e.g., display of available beds) information. (e.g., display of available beds) in a user-friendly manner" ("Totally agree": 0.6%/7, "Agree": 2.2%/24, "Somewhat agree":3.1%/34, "Somewhat disagree": 9.6%/105, "Disagree": 17.0%/186, "Totally disagree": 50.0%/548).

**Figure S2:** Responses to the question about the resulting improvements in collaboration from the measures shown in Figure 6 (max. 3 responses).

Note: The information is given in percent and the absolute number (n) in each case.

**Comparison of the university hospital with the non-university hospital**

The reasons for referral to the non-university hospital with which cooperation is most frequent are shown in Figure 3.

**Figure S3:** Responses to the question about reasons for admission to a non-university hospital (max. 3 responses).

Note: The information is given in percent and the absolute number (n) in each case.
